# Characteristics and Outcome of Abdominal Aortic Aneurysm in Emregncy Department; a 10-year Cross-sectional Study

**Published:** 2020-01-25

**Authors:** Mohammad Mehdi Forouzanfar, Fatemeh Barazesh, Behrooz Hashemi, Saeed Safari

**Affiliations:** 1 Emergency Department, Shohadye Tajrish Hospital, Shahid Behehsti University of Medical Sciences, Tehran, Iran.; 2 Proteomics Research Center, Shahid Beheshti University of Medical Sciences, Tehran, Iran.

**Keywords:** Aortic aneurysm, abdominal, abdominal pain, iliac aneurysm, outcome assessment

## Abstract

**Introduction:**

Abdominal aortic aneurysm (AAA, triple A) is one of the less common but important causes of abdominal pain. This study aimed to evaluate the characteristics and outcome of patients presenting to emergency department with triple A.

**Methods:**

In this retrospective cross-sectional study, all cases with confirmed triple A, who were presented to the emergency department of Shohadaye Tajrish Hospital, Tehran, Iran from 2006 to 2017 (10 years) were enrolled using census sampling method.

**Results:**

500 cases with the mean age of 68.11 ± 11.98 (25 - 94) years were studied (84% male). The mean duration of symptoms was 2.32 ± 9.58 months and mean aneurysmal size was 63.91 ± 20.08 mm. In 4 (0.8 %) cases, atrial fibrillation (AF) was found during cardiac monitoring. Patients stayed in the hospital for an average of 7.06 ± 6.32 days. Aneurysmal leak was seen in 130 (26%) cases based on abdominal computed tomography (CT) scan findings. 369 (73.8%) cases underwent aneurysmorrhaphy, 126 (25.2%) were treated with non-surgical approaches, and 5 (1%) underwent grafting. 104 (20.8%) died and 396 (79.2%) were treated successfully. Older age (p = 0.017), shock state at the time of presentation (p < 0.0001), leakage of aneurysm (p < 0.001), larger size of aneurysm (p = 0.024), and aneurysmorrhaphy (p < 0.001) were among the factors significantly associated with mortality.

**Conclusion:**

Based on the findings, the most frequent presenting symptom of patients was abdominal pain. The mortality rate of this series was 21% and older age, shock state, leakage of aneurysm, larger size of aneurysm, and performing aneurysmorrhaphy were among the factors significantly associated with mortality.

## Introduction

Abdominal aortic aneurysm is a progressive abnormal local dilatation due to gradual aorta wall weakness (1). It is defined as increase in the mean diameter of abdominal aorta by twice the standard error of mean, and has a prevalence rate of 0.5 to 7.2 percent in men and 1 to 1.3 percent in women (2, 3). Abdominal aortic aneurysm is usually asymptomatic until rupture (2). The risk of spontaneous rupture in these patients depends on the aortic diameter with rates of less than 0.5%, 1%, 11%, and 26% in cases with diameter less than 4 cm, 4-5 cm, 5-6 cm, and 6-7 cm, respectively (4). Nearly 50 to 90 percent of patients with aneurysmal rupture die before surgery, especially elderly men (5). Also, the postoperative mortality rate is more than forty percent (6). Prompt diagnosis and elective surgery would decrease the mortality rate to less than six percent (7). Postoperative 30-day mortality rate in elective cases is 5 to 8 percent (8). Considering the high mortality and morbidity rates in cases with abdominal aortic aneurysm and importance of early diagnosis to decrease the burden of the problem and noting the increase in frequency of aneurysm due to increased life longevity and higher rate of atherosclerotic diseases (9-14), this study aimed to evaluate the characteristics and outcome of patients with abdominal aortic aneurysm presenting to emergency department.

## Methods

***Study design and setting***

In this retrospective cross-sectional study, patients with abdominal aortic aneurysm presenting to emergency department of Shohadaye Tajrish Hospital, Tehran, Iran, from 2006 to 2017 (10 years) were studied. The study protocol was approved by ethics committee of Shahid Beheshti University of Medical Sciences (Ethics code: IR.SBMU.MSP.REC.1396.189). Researchers adhered to principles of Helsinki decleration regarding the ethical issues in biomedical studies.

***Participants***

All patients with confirmed abdominal aortic aneurysm, who had presented to the emergency department during the study period, were enrolled using census sampling method. Patients with an incomplete medical profile and those discharged against medical advice were excluded. There was not any age or sex limitation. 

***Data gathering***

Data were extracted from patients’ medical profiles using predesigned checklists containing demographic data (age, gender), presenting chief complaint, medical history, family history of aneurysm, characteristics of aneurysm (location, size), presence or absence of leakage based on computed tomography (CT) scan findings, and treatment approach, as well as outcome (mortality). A third year emergency medicine resident was responsible for data gathering. 

***Statistical analysis***

Data analysis was done using SPSS version 21.0 statistical software. Findings were presented as mean ± standard deviation or frequency (%) for numerical and categorical variables, respectively. The utilized tests were chi-square, independent-sample-t test, and Fisher’s exact test. P values less than 0.05 were considered statistically significant.

## Results

***Baseline characteristics of patients***

500 cases with the mean age of 68.11 ± 11.98 (25 - 94) years were studied (84% male). Table 1 shows the baseline characteristics of studied patients. Anatomical location of aneurysm was abdominal aorta in 468 (93.6%) cases, abdomino-thoracic aorta in 28 (5.6%) cases, and iliac artery in 4 (0.8%) cases. The most frequent chief complaint of patients at the time of presenting to ED was abdominal pain (73.8%). The mean duration of symptoms was 2.32 ± 9.58 months and mean aneurysmal size was 63.91 ± 20.08 mm. In 4 (0.8 %) cases, atrial fibrillation (AF) was found during cardiac monitoring. 

***Outcomes***

Patients stayed in the hospital for an average of 7.06 ± 6.32 days. Aneurysmal leak was seen in 130 (26%) cases based on abdominal CT scan findings. 369 (73.8%) cases underwent aneurysmorrhaphy, 126 (25.2%) were treated with non-surgical approaches, and 5 (1%) underwent grafting. 104 (20.8%) died and 396 (79.2%) were treated successfully. Older age (p = 0.017), shock state at the time of presentation (p < 0.0001), leakage of aneurysm (p < 0.001), larger size of aneurysm (p = 0.024), and aneurysmorrhaphy (p < 0.001) were among the factors significantly associated with mortality (table 2). 

## Discussion

Based on the findings, the most frequent presenting symptom of patients was abdominal pain. The mortality rate of this series was 21% and older age, shock state, leakage of aneurysm, larger size of aneurysm, and performing aneurysmorrhaphy were among the factors significantly associated with mortality. 

**Table 1:** Baseline characteristics of studied patients

**Variables**	**Frequency (%)**
**Gender**	
Male	412 (82.4)
Female	88 (17.6)
**Presenting cheif complaint**	
Abdominal pain	369 (73.8)
Shock state	30 (6.0)
Limb ischemia	40 (8.0)
Chest pain	22 (4.4)
Others	39 (7.8)
**Medical history**	
Cerebrovascular accident	44 (8.8)
Hypertension	258 (51.6)
Smoking	169 (33.8)
Dyslipidemia	57 (11.4)
Diabetes mellitus	50 (10.0)
Ischemic heart disease	173 (34.6)
Chronic kidney disease	23 (4.6)
Abdominal surgery	28 (5.6)
**Family history of aneurysm**	
No	497 (99.4)
Yes	3 (0.06)
**Location of aneurysm**	
Abdominal aorta	468 (93.6)
Iliac artery	4 (0.8)
Thoracic aorta	28 (5.6)
**Leakage of aneurysm**	
No	368 (79.2)
Yes	130 (20.8)

**Table 2:** Correlation between baseline characteristics of patients and mortality

**Variables**	**Survived (n = 396)**	**Died (n = 104)**	**P value**
**Gender**			
Male	329 (79.9)	83 (20.1)	0.435
Female	67 (76.1)	21 (23.9)	
**Age (year)**			
Mean ± SD	67.4 ± 11.8	70.7 ±12.3	0.017
Shock state*			
No	380 (80.9)	90 (19.1)	< 0.0001
Yes	16 (53.3)	14 (46.4)	
**Type of surgery**			
Not surgical	119 (94.4)	7 (5.6)	< 0.001
Aneurysmorrhaphy	272 (73.2)	97 (26.3)	
Grafting	5 (100.0)	0 (0.0)	
**Leakage of aeurysm**			
No	334 (90.8)	34 (9.2)	< 0.001
Yes	60 (46.2)	70 (53.8)	
**Location of aneurysm**			
Abdominal aorta	370 (79.1)	98 (20.9)	0.907
Iliac artery	3 (75.0)	1 (25.0)	
Thoracic aorta	23 (82.1)	5 (17.9)	
**Medical history**			
No	78 (80.4)	19 (19.6)	0.743
Yes	318 (78.9)	85 (21.1)	
**Duration of symptom (month)**			
Mean ± SD	1.9 ± 3.7	2.04 ± 5.4	0.881
**Aneurysm size (mm)**			
Mean ± SD	62.4 ±18.4	71.6 ± 21.9	0.024
**Duration of hospital stay**			
Mean ± SD	7.4 ± 5.8	5.7 ± 7.6	0.014

Data are presented as frequency (%) or mean ± standard deviation (SD).

*: At the time of presenting to emergency department.

Mirsharifi et al. (9) assessed 240 elderly patients using abdominal ultrasonography and incidentally found aorta aneurysm in 10% with mean diameter of 3.9 cm. 

Kuivaniem et al. (10) reported that smoking and family history are the most important risk factors for abdominal aortic aneurysm. They also reported that mortality rate ranged from 50 to 80 percent, whereas it was only 20 percent in our study.

Chabok et al. (11) assessed 50,000 female subjects from the general population and reported that 82 subjects had abdominal aortic aneurysm and also stated that smoking, hypertension, older age, ischemic heart disease (IHD), and stroke were its main risk factors. 

Tang et al. (12) assessed 15729 patients and reported that 5.5% had abdominal aortic aneurysm, which was more common among men, smokers, and those with dyslipidemia as shown in our study, especially about the effects of gender and smoking.

Yuan et al. (13) assessed 465 patients and reported hypertension, smoking, and dyslipidemia as the main risk factors for abdominal aortic aneurysm, which is similar to our results, especially regarding the first two factors. 

Giribono and colleagues (14) assessed nine cases with abdominal aortic aneurysm, 4 of which required endovascular surgery and all were successful. But in our study, twenty percent of the patients died, especially cases under aneurysmorrhaphy. 

Assessment of factors affecting the outcome is important for being able to predict the prognosis in patients with abdominal aortic aneurysm. In the current study, about 80% of the cases were discharged with good outome. 

Considering the factors significantly associated with mortality in this study (older age, shock state, leakage of aneurysm, larger size of aneurysm, and performing aneurysmorrhaphy), it seems that performing prophylactic abdominal ultrasonography in high risk subjects such as smokers and hypertensive patients could be helpful in detection of cases in younger age and with stable situation. Controlling the risk factors and planning for management before patients show symptoms and face a critical situation seems to be a logical strategy to reduce mortality.

## Limitations

Retrospective study design, missing data, not following the patients, and being a single center study were among the most important limitations of this study.

## Conclusion:

Based on the findings, the most frequent presenting symptom of patients was abdominal pain. The mortality rate of this series was 21% and older age, shock state, leakage of aneurysm, larger size of aneurysm, and performing aneurysmorrhaphy were among the factors significantly associated with mortality. 

## Author contribusions:

All the authors met the criteria of authorship based on the recommendations of the international committee of medical journal editors.
